# L´ostéomyélite aiguë à *Staphylocoque aureus* résistant à la méthicilline d´origine communautaire chez l´enfant: à propos de 15 cas

**DOI:** 10.11604/pamj.2021.39.84.12560

**Published:** 2021-05-28

**Authors:** Mohamed Ben Jemaa, Moez Trigui, Wassim Zribi, Emna Elleuch, Ameur Abid, Makram Koubaa, Basma Mnif, Zoubayer Ellouze, Kamel Ayedi, Adnène Hammemi, Mounir Ben Jemaa, Mohamed Zribi, Hassib Keskes

**Affiliations:** 1 Service de Chirurgie Orthopédique et Traumatologique, CHU Habib Bourguiba de Sfax, Sfax, Tunisie,; 2 Service des Maladies Infectieuses CHU Hédi Chaker de Sfax, Sfax, Tunisie,; 3 Laboratoire de Microbiologie, CHU Habib Bourguiba de Sfax, Sfax, Tunisie

**Keywords:** Ostéomyélite aiguë, *staphylocoque aureus*, résistance à la méthicilline, leucocidine de Panton et Valentine, pédiatrie, Acute osteomyelitis, staphylococcus aureus, methicillin resistance, Panton-Valentine leukocidin, pediatrics

## Abstract

La prise en charge de l´ostéomyélite aiguë devient plus délicate depuis l´émergence du Staphylocoque aureus résistant à la méthicilline d´origine communautaire. Nous avons recueilli les cas d´ostéomyélite aiguë dues à ce germe sur une période de 21 ans (Janvier 1995-Décembre 2016) et nous avons essayé d´analyser les particularités de cette entité pathologique. Notre série comporte 15 enfants, d´âge moyen 9 ans. Le membre inférieur était atteint dans tous les cas. Une notion de traumatisme local a été signalée dans 8 cas et une porte d´entrée cutanée a été trouvée dans 4 cas. Le mode de début était aigu dans 12 cas avec un tableau de pseudo-paralysie du membre atteint. Une staphylococcie pulmonaire avec des signes septico-pyohémiques ont été notés dans un cas. L´hémoculture était positive dans 8 cas. La recherche par PCR de la leucocidine de Panton et Valentine était pratiquée dans 1 cas avec un résultat positif. Tous ces patients ont eu un débridement chirurgical et une antibiothérapie empirique secondairement adaptée. L´évolution était bonne dans 8 cas et mauvaise dans les autres cas avec passage à la chronicité dans 6 cas et un cas de décès. Une fracture pathologique a été signalée dans 3 cas. La survenue d´une ostéomyélite à Staphylocoque aureus résistant à la méthicilline d´origine communautaire est péjorative. Connaitre ces infections en se basant sur des arguments cliniques et paracliniques est un enjeu important pour une prise en charge thérapeutique spécifique et rapide.

## Abstract (en)

The treatment of acute osteomyelitis is becoming more challenging since the emergence of community-acquired methicillin-resistant Staphylococcus aureus. We collected data on all patients with acute osteomyelitis caused by this germ over a period of 21 years (January 1995-December 2016) and we analyzed the peculiarities of this disorder. Our case series includes 15 children, with an average age of 9 years. All patients had affected lower limb. Local trauma was reported in 8 cases and skin carriage in 4 cases. Acute onset was reported in 12 cases associated with pseudo-paralysis of the affected limb. One patient had Staphylococcus aureus pulmonary infection with signs of septicopyemia. Blood culture was positive in 8 cases. In one case PCR assay for detection of Panton-Valentine leukocidin was performed with positive result. All these patients underwent surgical debridement and received secondarily adapted empirical antibiotic therapy. Outcome was good in 8 cases and poor in the other cases, with transition to a chronic state in 6 cases and one case of death. Pathological fracture was reported in 3 cases. Osteomyelitis cause by community-acquired methicillin-resistant Staphylococcus aureus is associated with a pejorative outcome. Recognizing the clinical and paraclinical signs of these infections is essential for a specific and early therapeutic management.

## Introduction

Plusieurs facteurs ont influencé les présentations cliniques et évolutives des ostéomyélites *aiguë*s (OMA). Parmi ces facteurs, interviennent les modifications du niveau socio-économique, l´amélioration de la prise en charge des infections par les antibiotiques et le changement de l´écologie bactérienne avec l´émergence de germes résistants comme *Staphylocoque aureus* résistant à la Méthicilline (SARM). Classiquement les infections à staphylocoques résistants aux antibiotiques sont nosocomiales. Depuis une vingtaine d´années, ont été décrites des infections communautaires à SARM. Il s´agit d´un germe résistant aux bêta-lactamines et sensible à la plupart des autres familles d´antibiotiques. Ce travail avait pour but de rapporter les particularités épidémiologiques, cliniques et paracliniques des OMA aux SARM communautaires (SARM-C) et leurs modalités thérapeutiques et évolutives.

## Méthodes

Nous avons revu rétrospectivement 105 cas d´OMA documentées microbiologiquement, colligés au service de chirurgie orthopédique et traumatologique de Sfax de la Tunisie, durant une période de 21 ans (de janvier 1995 à décembre 2016). Parmi ces OMA, nous nous sommes intéressés aux OMA dues à SARM-C.

### Différents critères ont été recherchés

**Données démographiques et d´anamnèse:** origine géographique, âge, sexe, antécédents, traitement reçu avant l´hospitalisation.

**Données cliniques:** douleur, fièvre, porte d´entrée cutanée, signes inflammatoires locaux, localisations infectieuses associées.

**Données biologiques:** leucocytose sanguine, protéine C-Reactive (CRP), vitesse de sédimentation (VS).

**Données d´imagerie:** radiographie standard, échographie.

**Données bactériologiques:** hémocultures, prélèvement chirurgical de l´abcès osseux.

**Données de la biologie moléculaire:** recherche des toxines: la leucocidine de Panton et Valentine (PVL) par la technique de PCR.

**La conduite thérapeutique:** (antibiothérapie, immobilisation et durée du traitement).

**L´évolution sous traitement et à distance:** (complications, résultats fonctionnels et anatomiques).

## Résultats

**Figure 1:**
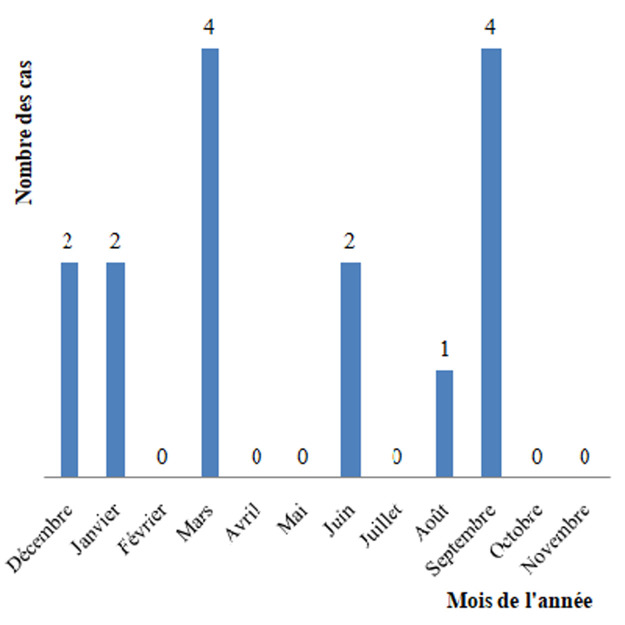
répartition mensuelle des cas d´ostéomyélites aiguës à SARM-C

**Résultats épidémiologiques:** le SARM-C était isolé dans 15 cas parmi 105 cas d´OMA à germe identifié soit un pourcentage de 14%. Le reste des germes étaient le *S. areus* sensible à la Méthicilline (SAMS) (77%), *Streptocoque* (6%), *Haemophilus influenzae* (2%) et *Pseudomonas* (1%). L´âge moyen était de 9 ans (2 à 16 ans) avec une nette prédominance masculine constatée (11 garçons et 4 filles). Le niveau socioéconomique était moyen dans 9 cas et bon dans 6 cas. La représentation mensuelle des cas est représentée par la Figure 1.

**Résultats cliniques:** le membre inférieur était atteint dans tous les cas. L´atteinte était monofocale dans 14 cas et bifocale avec atteinte simultanée de l´extrémité supérieure du fémur et celle du péroné dans 1 cas. L´atteinte des métaphyses des os longs était constatée dans 13 cas et celle des os plats dans 2 cas. Les différentes localisations étaient: l´extrémité supérieure du tibia (4 cas), l´extrémité supérieure du fémur (2 cas), l´extrémité inférieure du fémur (3 cas), l´extrémité inférieure du tibia (2 cas), l´extrémité inférieure du péroné (2 cas), l´extrémité supérieure du péroné (1 cas) et l´os iliaque (2 cas). Une notion de traumatisme était rapportée dans 8 cas. Un seul enfant avait un antécédent de cardiopathie congénitale. Aucun antécédent pathologique n´a été trouvé chez les autres enfants.

**Figure 2:**
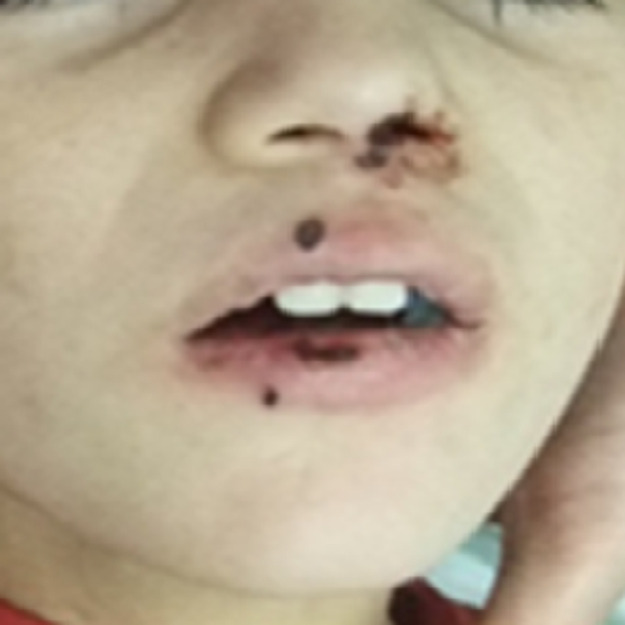
lésions d´impétigo entre l´orifice nasal et buccal avec des lésions cutanéomuqueuses satellites

Le mode de début était aigu dans 12 cas et subaigu dans les autres cas. Un retard de consultation était noté dans 3 cas (2 cas d´OMA abâtardies par une antibiothérapie prise à l´aveugle et le cas de l´atteinte bifocale). Une fièvre était constatée dans 14 des cas (93%). Tous les patients avaient un tableau de pseudo-paralysie du membre atteint avec une impotence fonctionnelle totale, une douleur osseuse exquise et des signes inflammatoires locaux. Un mauvais état général avec un teint infecté était constaté dans 5 cas (33%). Des signes de gravité à type d´état de choc septique avec une staphylococcie pulmonaire étaient trouvés dans un cas. Une porte d´entrée cutanée était notée dans 4 cas (27%). Elle était à type d´impétigo commun dans un cas (Figure 2).

**Données biologiques:** un syndrome inflammatoire biologique était retrouvé dans tous les cas avec des valeurs moyennes de CRP à 203mg/l et de VS à 60mm à la première heure. Le taux des leucocytes était normal dans 9 cas (60%). Une hyperleucocytose a été trouvée dans 6 cas (40%). Aucune leucopénie n´a été trouvée.

**Données microbiologiques:** l´examen microbiologique du prélèvement peropératoire a montré un SARM-C chez tous les patients. Le même germe était isolé dans l´hémoculture de 8 cas (53%). L´antibiogramme des six derniers cas a objectivé une résistance à des molécules différentes des bêta-lactamines. Cette résistance est illustrée dans le Tableau 1.

**Tableau 1:** profil de résistance des SARM-C aux antibiotiques

Antibiotiques	Pourcentage de résistance (%)
Kanamycine	40%
Amikacine	40%
Tétracyclines	40%
Pristinamycine	7%
Erythromycine	7%
Pefloxacine	7%
Ofloxacine	7%
Norfloxacine	7%
Ciprofloxacine	7%
Acide fucidique	40%

**Données de la biologie moléculaire:** la recherche par PCR d´ADN de la PVL a été effectuée une seule fois avec un résultat positif.

**Données de l´imagerie initiale:** la radiographie standard initiale du membre atteint était sans anomalies dans tous les cas. L´échographie initiale, était faite pour 14 patients. Elle a mis en évidence un abcès sous périosté dans 9 cas, non rompu (6 cas) et rompu dans les parties molles (3 cas). Une infiltration des parties molles sans décollement périosté était notée dans 1 cas. Quatre patients, ayant une échographie initiale normale, ont développé un abcès sous périosté secondairement. Une tomodensitométrie thoraco-abdomino-pelvienne a été pratiquée un patient ayant une OMA de l´aile iliaque associée à une atteinte pulmonaire nécrosante. Elle a objectivé des collections au contact de l´os iliaque fusant dans les muscles fessiers et la loge antérieure de la cuisse.

**Traitement:** une antibiothérapie parentérale probabiliste associant 2 molécules, a été administrée pour tous les patients: oxacilline ou l´amoxicilline/acide-clavulanique + gentamycine (12 cas) et fosfomycine + cefotaxime (3 cas). Cette antibiothérapie a été adaptée après résultats microbiologiques pour 12 patients. Elle a fait appel à des molécules diverses telle que l´association des glycopeptides (vancomycine ou teichoplanine) à la rifampicine (4 cas). La durée moyenne du traitement parentéral était de 3 semaines (1 semaine-16 semaines). Un relais par voie orale était systématique dans tous les cas. Il a fait appel à l´association de la rifampicine à la sulfaméthoxazole/thriméthoprime dans 6 cas. La durée moyenne du traitement oral était de 8 semaines (3 semaines - 18 semaines).

Tous les patients étaient opérés dès la visualisation paraclinique d´un abcès sous périosté dans 14 cas et en l´absence sa mise en évidence dans 1 cas. Ils ont eu une chirurgie de débridement avec un drainage de l´abcès sous périosté, une trépanation corticale et un lavage diaphysaire. L´exploration chirurgicale a montré la présence d´un abcès périosté non rompu dans 9 cas, rompu dans 6 cas, une pandiaphysite dans 3 cas et une ostéo-arthrite dans 2 cas. Tous les cas ont eu une immobilisation par attelle plâtrée. La durée moyenne d´hospitalisation était de 4 semaines.

**Figure 3:**
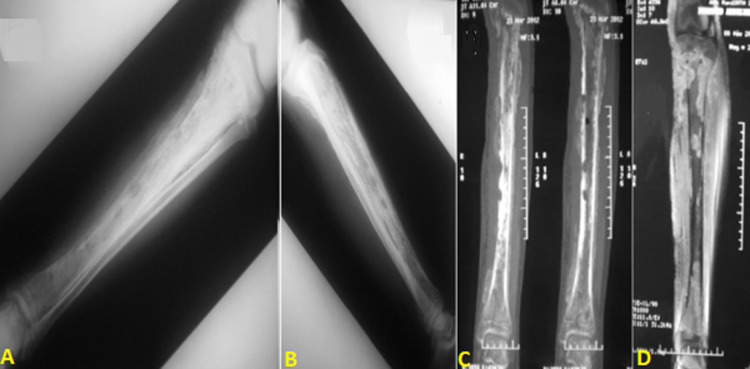
radiographie standard de la jambe (A,B) complétée par une tomodensitométrie (C) et une IRM (D) pour une ostéomyélite de l´extrémité supérieure du tibia compliquée d´une pandiaphysite

**Evolution post-opératoire précoce:** les suites post-opératoires précoces étaient marquées par: le décès du cas compliqué de staphylococcie pulmonaire avec défaillance multi-viscérale ; la persistance de la fièvre et d´un mauvais état local dans 5 cas. D´où la nécessité d´une reprise chirurgicale pour laquelle une extension pandiaphysaire de l´infection a été constatée en peropératoire (Figure 3); une rémission clinique retardée dans les autres cas.

**Figure 4:**
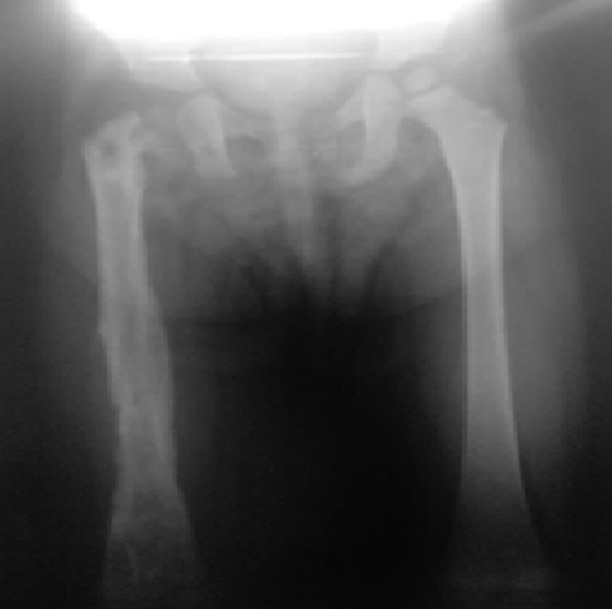
radiographie standard du fémur droit prenant le fémur controlatéral: ostéomyélite aiguë du col du fémur droit compliquée d´une pandiaphysite chronique avec raccourcissement fémoral et varisation du col

**Figure 5:**
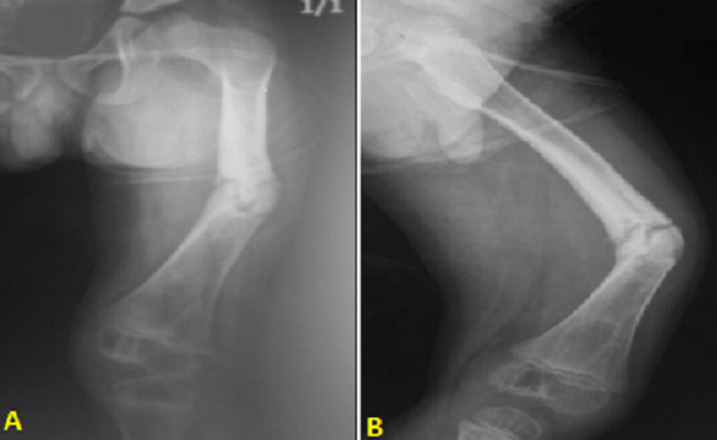
radiographie standard du fémur gauche (A,B): ostéomyélite aiguë de l´extrémité inférieure du fémur compliquée d´une pandiaphysite chronique avec fracture récidivante et raccourcissement par télescopage du foyer fracturaire

**Evolution post-opératoire tardive:** les suites post-opératoires tardives étaient favorables dans 8 cas (53%) avec reprise d´une marche normale à 6 mois. Un passage à la chronicité a été constaté dans 6 cas (40%). Il était caractérisé par un handicap fonctionnel majeur, une persistance du syndrome inflammatoire biologique et une pandiaphysite chronique à la radiographie de contrôle (Figure 4). Elle était compliquée d´une fracture sur os pathologique dans 3 cas avec raccourcissement du membre par télescopage du foyer de fracture (Figure 5).

## Discussion

Le *Staphylocoque aureus* résistant à la méthicilline est souvent responsable d´infections nosocomiales. Toutefois, depuis quelques années, il y a eu une émergence de souches de SARM-C. Ce germe a été décrit pour la première fois en 1983 au Michigan. Le SARM est considéré communautaire lorsqu´il répond aux critères suivants [1]: l´isolement de SARM chez un patient en soins ambulatoires ou dans les premières 48 heures de l´admission dans un centre hospitalier; absence d´antécédents d´infection ou de colonisation à SARM; absence d´hospitalisation, d´admission dans un établissement de longue durée, de dialyse ou de chirurgie dans l´année précédente; absence de cathéters permanents ou d´autres appareils médicaux traversant la peau. La résistance de SARM est due à l´acquisition d´un gène appelé mecA. Le gène mecA est localisé sur la cassette chromosomale staphylococcique (CCS) responsable de la régulation de l´expression du mecA. Jusqu´à maintenant, cinq types majeurs de CCS ont été identifiés (I-V). Les types I, II et III sont ceux retrouvés le plus fréquemment dans les isolats du SARM-N. Ils confèrent une résistance à d´autres antibiotiques en plus des bêta-lactamines. Les types IV et V sont plus petits, caractérisent plutôt les SARM-C et ne confèrent pas de résistance à d´autres antibiotiques que les bêta-lactamines [2].

Le SARM-C produit souvent la toxine de PVL responsable de tableaux cliniques sévères. La présence de gène codant pour la PVL est quasi-pathognomonique du SARM-C. Cette toxine a une action cytotoxique au niveau des leucocytes humains ce qui pourrait expliquer l´absence d´hyperleucocytose voire la leucopénie constatée en cas d´infection à SARM-C. Elle est responsable de tableaux cliniques sévères notamment des infections nécrosantes de la peau et des tissus mous, des pneumonies nécrosantes et des ostéomyélites sévères [3]. Malgré son action cytotoxique importante, elle est dépourvue d´activité super-antigénique donc elle ne peut être neutralisé par des immunoglobulines spécifiques [4]. Une augmentation de fréquence des infections ostéo-articulaires aigues a été rapportée par Arnold et al qui ont constaté une augmentation de l´incidence de 2.6 à 6 pour 1000 admissions entre 2000 et 2004 [5]. Cette augmentation était synchrone à l´émergence des infections à SARM-C qui est passée de 4 à 40% durant la même période alors que l´incidence des infections à *S. aureus* sensible à la Méthicilline (SASM) est restée constante [6]. Des situations épidémiques ont été décrites en particulier au Texas en 2005 où plus de 70% des souches de *S. aureus* d'origine communautaire étaient des SARM [7]. La dissémination épidémique de ces souches semble être favorisée par la promiscuité (crèches, communautés de sportifs, militaires ou prisonniers) [8]. Plus tard, l'émergence des SARM-C a été observée en maternité ou dans des unités de néonatologie [9,10]. La distinction entre SARM-N et Les SARM-C risque de devenir difficile puisque les SARM-N peuvent diffuser dans la communauté et les SARM-C peuvent disséminer en milieu hospitalier [11]. Le Tableau 2 représente les principales différences entre le SARM-C et le SARM-N. Actuellement, le SARM-C est responsable de la majorité des infections ostéo-articulaires aigues dans certaines régions [5,6,12,13]. Dans notre série nous avons observé que les OMA à SARM-C sont relativement rares (14%), bien que leur incidence est en train d´augmenter.

**Tableau 2:** principales différences entre le SARM-C et le SARM-N

Caractéristiques	SARM-N	SARM-C
Population cible	Sujets âgés soins de santé	Enfants, jeunes utilisateurs de drogues
Porte d´entrée	Site opératoire+	Infection cutanées multiples et récurrentes non invasives (furonculose, impétigo)
Marqueurs moléculaires	SCCmec I-III	SCCmec IV, V
Résistance aux antibiotiques	Multi résistance	Résistance presque unique aux bêta-lactamines
Présence de toxines PVL	rare	+

Kaplan et al ont montré que parmi 117 patients ayant eu une infection invasive à SARM-C, 46,1% présentaient une OMA [7]. Par contre, parmi les 76 patients ayant une infection à SASM, 36,8% seulement présentaient une OMA. Les foyers multiples d'ostéomyélites sont plus fréquents lorsqu'un SAMR-C est en cause [14]. Martinez-Arguilar *et al*. ont démontré que les durées de la fièvre et d´hospitalisation sont plus longues lorsqu'il s'agit d'un SARM-C plutôt que SASM [13]. La sévérité du tableau clinique des infections à SARM-C justifie l´intérêt de les reconnaitre en se basant sur des éléments cliniques et paracliniques et orienter le laboratoire de bactériologie à la recherche de PVL. Ces infections peuvent être évoquées devant les éléments suivants [12]: coexistence ou antécédents récents d'infection cutanée suppurée, à fortiori celles-ci est d'allure primitive ou s'il s'agit d'une forme familiale et/ou récidivante (95% des furonculoses sont dues à des souches VPL+), début brutal, d'emblée bruyant, avec une fièvre élevée et une impotence fonctionnelle marquée, une évolution rapide vers un tableau de sepsis sévère voire de choc septique, une discordance entre une l´absence d´hyperleucocytose voire une leucopénie et une élévation marquée de la CRP, la précocité des signes radiologiques. Le choix des antibiotiques pour le traitement empirique de l´OMA chez les enfants doit toujours englober dans son spectre le *S. aureus*. Anciennement, l´oxacilline était l´antibiotique de choix en association avec d´autres molécules anti-staphylococciques. Actuellement, une connaissance de l´écologie bactérienne locale et notamment le pourcentage de SARM-C est nécessaire pour pouvoir proposer une antibiothérapie empirique qui doit comporter au moins une molécule à action anti-PVL.

Parmi les molécules d´antibiotiques qu´on peut utiliser pour traiter une OMA à SARM-C, on trouve la clindamycine qui parait la plus indiquée car en plus de son action sur le SARM, elle diminue la production de PVL [1,15]. Des résistances à la clindamycine peuvent exister et doivent être recherchées. Dans la plupart des régions, 90% ou plus des isolats SARM-C sont sensibles à la clindamycine [16]. Lorsque le taux de résistance de *S. aureus* à la clindamycine dans la communauté dépasse 10% à 15%, la clindamycine n'est plus recommandée pour le traitement empirique [15]. Dans ces cas, on peut utiliser d´autres antibiotiques comme la Vancomycine. Toutefois, le recours à la vancomycine doit être prudent du fait de sa nephrotoxicité en plus des cas de résistances à la vancomycine devenus plus fréquents. D´autres alternatives thérapeutiques existent, telles que le Linézolide dont l´usage pédiatrique est approuvé. Il s´agit d´une molécule intéressante pour le relais par voie orale pour l´OMA à SARM résistants à la clindamycine. Dans notre série, d'autres options thérapeutiques pour les OMA à SARM-C ont montrés leurs efficacités telles que la Teichoplanine et la Rifampicine. Ces formes graves nécessiteront en plus une antibiothérapie intraveineuse puis orale plus prolongée [17]. Une bithérapie parentérale au moins jusqu'à l'obtention d'une apyrexie durable, en l´absence de complications post-opératoires, parait nécessaire [18].

La chirurgie occupe une place importante dans le traitement. Elle consiste à l´évacuation d´un abcès sous-périosté et intra-médullaire [15]. Elle doit être posée le plus rapidement possible dès qu'il existe des signes d'abcèdation en imagerie et dès qu'on a la preuve ou de fortes présomptions qu'il s'agit d'une souche PVL+. I1 ne faudra pas hésiter à répéter les interventions chirurgicales devant une mauvaise évolution post-opératoire [18]. En post-opératoire précoce, La persistance ou la réapparition de la fièvre incite à chercher une localisation secondaire ostéo-articulaire, pulmonaire, péricardique ou une résistance des germes aux antibiotiques prescrits. Bocchini *et al*. signalent que 15% des enfants atteints d´OMA causée par les souches de *S. aureus* PVL+ disposent de plusieurs sites d´infection [15,17]. Des complications particulières à ces formes d´infection à germes PVL+ à type de thrombophlébites doivent être recherchées [12]. Ces complications semblent être plus fréquentes en cas d´OMA à SARM-C [15,19,20]. Dans une série de 70 cas d´OMA dont seulement 44 cas avaient une identification microbiologique, Bouchoucha *et al*. ont rapporté 7 cas de thrombophlébites secondaires à un SARM-C dans 3 cas parmi 6 (50%) et à un SAMS dans 4 cas parmi 33 (12,1%) [17]. En outre, l´évolution vers la chronicité est plus susceptible d´être trouvée au moment du suivi avec des isolats PVL+ que des isolats PVL- [15]. Le Tableau 3 représente les principales différences cliniques, paracliniques et évolutives entre le SARM-C et le SAMS.

**Tableau 3:** principales différences entre SARM-C et SASM

Caractéristiques	SAMS	SAMR-C
Tropisme ostéo-articulaire	+	+
Atteinte multifocale	+/-	+
Niveau socio-économique	Bas	Bon ou moyen
Sévérité du tableau clinque	+/-	+
Sepsis	+/-	+
Hémocultures	+/-	+
Syndrome inflammatoire biologique	Hyperleucocytose+, VS+, CRP +	Normo-leucocytose +, Hyperleucocytose +, leucopénie +/-, VS+, CPR+
Lésions radiologiques	retardées	précoces
Durée d´hospitalisation	+	+
Complications	+/-	Recollection, complications thromboemboliques et chronicité+

## Conclusion

Les ostéomyélites à *Staphylocoque aureus* résistant à la méthicilline d´origine communautaire sont graves et de plus en plus fréquentes dans le monde. La recherche de la leucocidine de Panton et Valentine par PCR permet un diagnostic précoce et une adaptation rapide de l´antibiothérapie empirique. En l´absence de cette toxine et avant les résultats microbiologiques, le praticien doit adapter rapidement le traitement en se basant sur des arguments cliniques et paracliniques. Seule une prise en charge médico-chirurgicale optimale permet d´éviter les séquelles d´handicap majeures et les évolutions fatales.

### Etat des connaissances sur le sujet

- L´ostéomyélite est une urgence ortho-pédiatrique dont le pronostic est étroitement liée non seulement à la précocité de la prise en charge mais aussi à l´efficacité de l´antibiothérapie administrée;
- Le Staphylocoque aureus est le germe le plus fréquemment en cause. Il est souvent sensible à la méthicilline. L´émergence des souches résistantes à la méthicilline d´origine communautaire est de plus en plus fréquente dans le monde;
- Le Staphylocoque aureus résistant à la méthicilline d´origine communautaire est caractérisé par la production d´une toxine appelée la Leucocidine de Panton et Valentine qui aggrave la sévérité du tableau.

### Contribution de notre étude à la connaissance

- Le praticien doit suspecter le Staphylocoque aureus résistant à la méthicillie d´origine communautaire devant toute ostéomyélite aiguë en se basant sur des arguments cliniques et para-cliniques (antécédents ou présence de lésions cutanées suppurées, gravité du syndrome infectieux, atteinte pulmonaire, multifocalité de l´atteinte ostéomyélitique, forte élévation de la CRP avec absence d´hyperleucocytose);
- Le recours à la recherche de la leucocidine de Panton et Valentine par PCR semble utile car elle fournit un résultat rapide permettant ainsi une adaptation précoce l´antibiothérapie;
- Le spectre de l´antibiothérapie initiale probabiliste doit couvrir le Staphylocoque aureus résistant à la méthicillie d´origine communautaire dès qu´on suspecte ce germe afin de prévenir les complications précoces et tardives secondaire dues à une prise en charge tardive et inadaptée.
